# *Bacteroidales* Secreted Antimicrobial Proteins Target Surface Molecules Necessary for Gut Colonization and Mediate Competition *In Vivo*

**DOI:** 10.1128/mBio.01055-16

**Published:** 2016-08-23

**Authors:** Kevin G. Roelofs, Michael J. Coyne, Rahul R. Gentyala, Maria Chatzidaki-Livanis, Laurie E. Comstock

**Affiliations:** Division of Infectious Diseases, Brigham and Women’s Hospital, Harvard Medical School, Boston, Massachusetts, USA

## Abstract

We recently showed that human gut *Bacteroidales* species secrete antimicrobial proteins (BSAPs), and we characterized *in vitro* the first such BSAP produced by *Bacteroides fragilis*. In this study, we identified a second potent BSAP produced by the ubiquitous and abundant human gut species *Bacteroides uniformis*. The two BSAPs contain a membrane attack complex/perforin (MACPF) domain but share very little sequence similarity. We identified the target molecules of BSAP-sensitive cells and showed that each BSAP targets a different class of surface molecule: BSAP-1 targets an outer membrane protein of sensitive *B. fragilis* strains, and BSAP-2 targets the O-antigen glycan of lipopolysaccharide (LPS) of sensitive *B. uniformis* strains. Species-wide genomic and phenotypic analyses of *B. fragilis* and *B. uniformis* showed that BSAP-producing strains circumvent killing by synthesizing an orthologous nontargeted surface molecule. The BSAP genes are adjacent to the gene(s) encoding their target replacements, suggesting coacquisition. Using a gnotobiotic mouse competitive-colonization model, we found that the BSAP surface targets are important for colonization of the mammalian gut, thereby explaining why they are maintained in sensitive strains and why they were replaced rather than deleted in BSAP-producing strains. Using isogenic BSAP-producing, -sensitive, and -resistant strains, we show that a BSAP-producing strain outcompetes a sensitive strain but not a resistant strain in the mammalian gut. Human gut metagenomic datasets reveal that BSAP-1-sensitive strains do not cooccur with BSAP-1-producing strains in human gut microbiotas, further supporting the idea that BSAPs are important competitive factors with relevance to the strain-level composition of the human gut microbiota.

## IMPORTANCE

We know relatively little about the ecology of the human intestinal microbiota and the combination of factors that dictate which strains and species occupy an individual’s gut microbial community. Interference competition, mediated by bacterial factors that directly harm other members, is beginning to be appreciated as important in contributing to species- and strain-level dynamics of abundant gut bacteria. Here, we show that gut *Bacteroidales* secrete antimicrobial proteins (BSAPs) that antagonize strains of the same species. We show that BSAPs target molecules of sensitive cells that are important for gut colonization and therefore are maintained in sensitive cells. In an experimental animal model of gut colonization, a BSAP-1-producing strain antagonized and outcompeted an isogenic sensitive strain. Furthermore, metagenomic analyses showed that BSAP-1-producing and -sensitive strains are not found together in human gut microbiotas. These data suggest that BSAPs are strong ecological drivers shaping the strain-level composition of gut communities.

## INTRODUCTION

Human intestines harbor unique microbial communities containing hundreds of individual bacterial strains that compete for resources and occupation of intestinal niches (1, 2). Variation in the structure and function of these microbial communities affects many aspects of host biology, including nutrition (3, 4), metabolism (5), immune function (6), and susceptibility to infection (7). Thus, the factors that shape human-associated microbial communities are the subject of great scientific interest (8).

In addition to the importance of host and dietary factors in shaping the gut microbiota, we are beginning to more fully appreciate the role of microbe-microbe interactions in shaping these communities. Studies have analyzed by-product syntrophy among gut microbes, where one bacterium metabolizes the waste products of a phylogenetically distant species (9, 10). Other studies have shown that pathogens can benefit by utilizing sugar moieties of host glycans liberated by gut symbionts (11, 12). In addition, polysaccharide breakdown products have been shown to serve as public goods, mediating beneficial interactions among closely related gut species (13), in some cases benefitting both producer and utilizer (14). However, for ecosystems with high species diversity such as the gut microbiota, an abundance of cooperative interactions is predicted to result in a fragile community structure where small perturbations are magnified by codependent feedback loops (15). Modeling suggests that competitive interactions limit the systemic importance of any one species, leading to a stable community structure.

Two mechanisms of competition are prominent in bacterial communities: exploitative competition, where members compete for shared nutrients and resources, and interference competition, in which a member directly harms a competitor, often through the production of an antimicrobial molecule (16, 17). Exploitative competition is likely one of the most important ecological factors in determining which members stably colonize the mammalian gut. When exploitative competition between members is high, interference competition is likely to be very important in providing an advantage to a member able to antagonize its competitor. Several bacterially produced antimicrobial factors have been studied in the gut ecosystem, including those that require microbe-microbe contact, such as type VI secretion systems (T6SSs) (18–20), and those that are actively secreted or released from bacteria, such as phage (21), inhibitory metabolites (22), bacteriocins (23, 24), and antimicrobial proteins (25, 26).

A few studies have addressed the ecological effects of secreted antimicrobial molecules on the composition of the gut microbiota. One of the first animal studies to analyze the effects of secreted antimicrobial molecules *in vivo* demonstrated that colicin E2 of *Escherichia coli* likely promotes rather than eliminates microbial diversity in the gut due to the structured nature of the ecosystem (26). A recent study showed that production of colicin Ib conferred a competitive advantage to *Salmonella enterica* serovar Typhimurium over *E. coli* only in the inflamed gut, where both the colicin and the receptor of sensitive *E. coli* strains are upregulated (27). Another recent study analyzed bacteriocin production by enterococci. In this system, enterococci expressing bacteriocin 21 replaced indigenous enterococci and outcompeted *Enterococcus faecalis* lacking the bacteriocin (23). Although these were important studies demonstrating ecological effects of secreted antimicrobial molecules, some with significant implications for infectious diseases and their prevention and treatment, they addressed lower-abundance members of the gut microbiota.

*Bacteroidales* species are highly abundant in the gut microbiota (28) and temporally stable, with individual strains maintained for decades and horizontally transferred from parents to children (1). We recently identified a *Bacteroidales*-secreted antimicrobial protein (BSAP-1) of *Bacteroides fragilis* that antagonizes *in vitro* a subset of *B. fragilis* strains lacking BSAP-1 (25). BSAP-1 contains a membrane attack complex/perforin (MACPF) domain related to the cholesterol-dependent cytolysin (CDC) domain of Gram-positive pore-forming toxins (29). In this report, we identify a second BSAP (BSAP-2) with a MACPF domain, produced by *Bacteroides uniformis*. We provide comprehensive analyses of both BSAP-1 and BSAP-2 and identify their cognate targets in sensitive strains and mechanisms of resistance in BSAP-producing strains. Studies in gnotobiotic mice combined with analyses of human gut metagenomes suggested that BSAPs mediate interference competition and contribute to the strain-level composition of *Bacteroidales* in the human gut.

## RESULTS

### Identification of BSAP-2 in *B. uniformis.*

**FIG 1:**
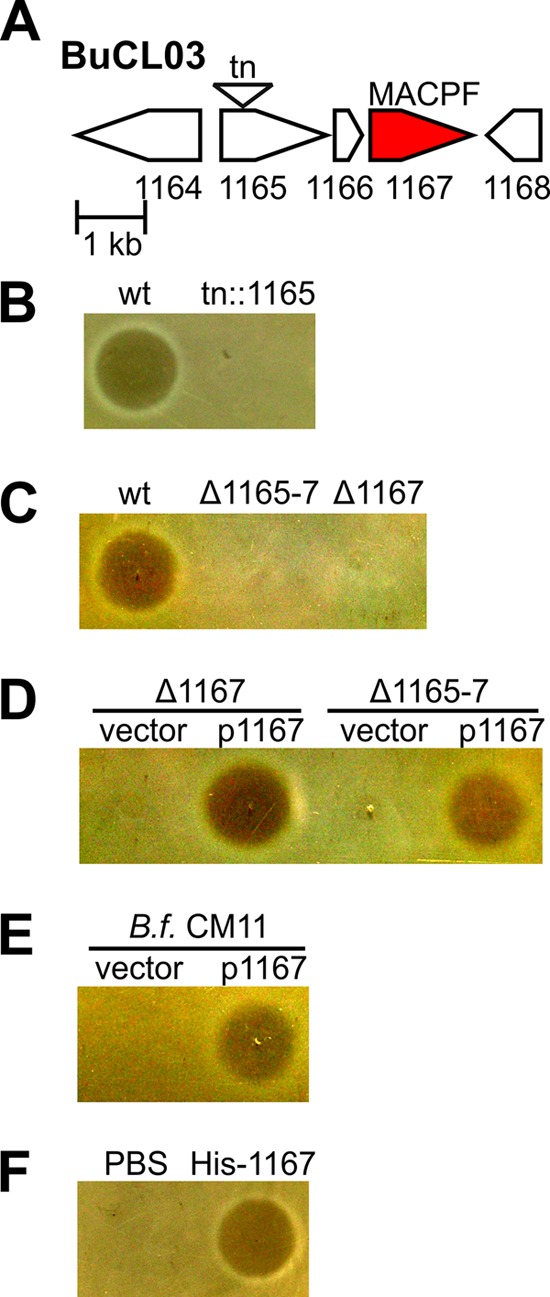
Identification of BSAP-2. (A) ORF map of a *B. uniformis* CL03T00C23 (BuCL03) region showing a transposon (tn) insertion resulting in loss in antimicrobial activity against *B. uniformis* 8492 (Bu8492). (B to F) Agar overlay assays showing growth or inhibition of Bu8492 by (B) BuCL03 wild type (wt) and transposon mutant *tn*::1165. (C) The BUCL03 wt and deletion mutants of HMPREF1072_1165-7 (Δ1165-7) or HMPREF1072_1167 (Δ1167), (D) BuCL03 Δ1165-7 and Δ1167 mutants containing empty vector (vector) or plasmid-expressing HMPREF1072_01167 (1167), (E) *B. fragilis* (*B.f.*) CM11 with empty vector or 1167, (F) and a phosphate-buffered saline (PBS) control or purified His-tagged 1167 (His-1167).

To broaden our analysis of BSAP production in the gut *Bacteroides*, we analyzed other *Bacteroides* species for the production of secreted antimicrobial molecules against species-matched strains. We previously demonstrated that 7/10 *B. fragilis* strains tested produced secreted antimicrobial molecules (25). In this study, we found that 7/17 *B. vulgatus*, 2/10 *B. dorei*, 2/6 *B. uniformis*, 0/9 *B. cellulosilyticus*, and 0/6 *B. stercoris* strains tested secreted antimicrobial molecules detected using an agar overlay assay. For this study, we focused on the secreted antimicrobial molecule produced by *B. uniformis* CL03T00C23 (BuCL03) as it potently inhibits the growth of the *B. uniformis* ATCC 8492 (Bu8492) type strain. Transposon mutants of BuCL03 were screened for loss of inhibitory activity, and a mutant with an insertion in HMPREF1072_01165 (1165) lost the ability to inhibit Bu8492 (Fig. 1A and B). 1165 is the first gene in a three-gene operon that includes HMPREF1072_01167 (1167), which encodes a MACPF domain protein (Fig. 1A). As BSAP-1 of *B. fragilis* also contains a MACPF domain, we predicted that 1167 may encode the antimicrobial activity. We made a deletion removing all three genes together (Δ1165, Δ1166, and Δ1167 [Δ1165-1167]) and a second deletion where only the MACPF-encoding gene was deleted (Δ1167). Both mutants lost killing activity (Fig. 1C). Expression of 1167 in *trans* restored antimicrobial activity to both mutants (Fig. 1D), demonstrating that 1167 is the only gene of this operon necessary for the antimicrobial activity. To further confirm that 1167 encodes the antimicrobial activity, we expressed this gene in *B. fragilis* CM11 and found that it confers upon this heterologous species the ability to inhibit Bu8492 (Fig. 1E). To test if the protein product of 1167 alone is able to inhibit sensitive Bu8492, recombinant His-1167 was purified from *E. coli*. The purified protein inhibited the growth of Bu8492 in agar overlays, definitively identifying this protein as the antimicrobial molecule (Fig. 1F). Based on these collective findings, the protein encoded by HMPREF1072_01167 is designated BSAP-2. Although both BSAP-1 and BSAP-2 contain MACPF domains and share critical conserved MACPF domain residues (30) shown to be necessary for BSAP-1 activity (25) (see Fig. S1 in the supplemental material), they are only 45% similar to each other (see Fig. S2 and Table S2).

### Specific O-ag of LPS is required for BSAP-2 sensitivity.

**FIG 2:**
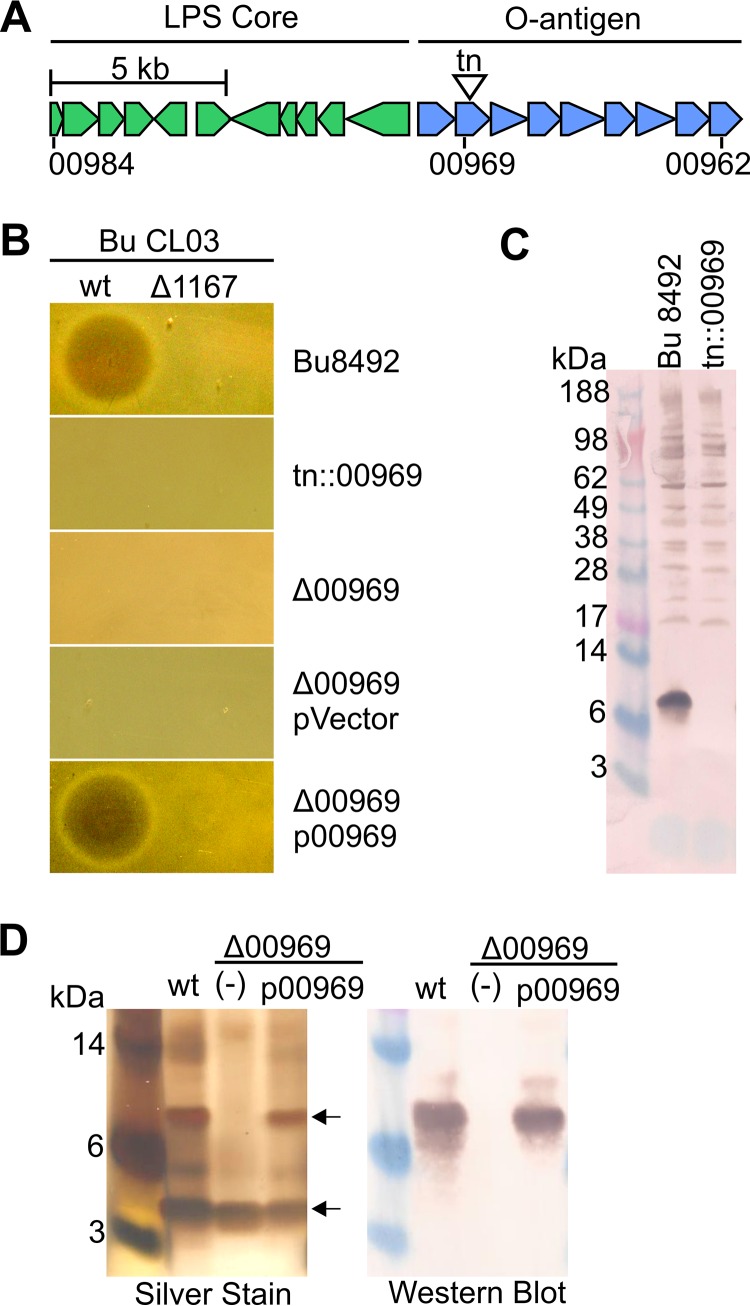
BSAP-2 targets the O-ag of *B. uniformis* 8492. (A) Genetic region of Bu8492 encoding predicted LPS core and O-antigen biosynthesis loci. A transposon insertion into BACUNI_00969 causing BSAP-2 resistance is indicated. (B) Agar overlay assays showing sensitivity of Bu8492 strains to BuCL03 wt or the mutant lacking the gene encoding BSAP-2 (Δ1167). Overlay strains include BACUNI_00969 deletion mutant (Δ00969), Δ00969 with an empty vector (pVector), or BACUNI_00969 (p00969) in *trans.* (C) Western blot of whole-cell lysates of Bu8492 or *tn*::00969 probed with antiserum raised to Bu8492 and adsorbed against the *tn*::00969 mutant. (D) Silver stain of purified LPS from Bu8492 or Δ00969 with vector alone (−) or p00969 in *trans*. (E) Same as panel D except probed with the adsorbed serum used as described for panel C.

The mechanism of BSAP sensitivity and resistance has not been shown, but as BSAP-1 and BSAP-2 target only a subset of species-matched strains, we predicted that BSAPs target surface molecules not present in producer strains. To identify the target of BSAP-2 in Bu8492, a transposon mutant bank was serially cocultured on agar plates with BSAP-2-producing BuCL03 to enrich for a resistant population. This enrichment resulted in the identification of a BSAP-2-resistant transposon mutant with an insertion in BACUNI_00969 (Fig. 2A and B). A mutant with a clean deletion of this gene was also resistant to BSAP-2 killing, with BSAP-2 sensitivity restored when BACUNI_00969 was provided to the mutant in *trans* (Fig. 2B). BACUNI_00969 encodes a predicted glycosyltransferase and is the second gene of a predicted O-antigen (O-ag) biosynthesis locus spanning BACUNI_00970 to BACUNI_00962 (BACUNI_00970-00962) (Fig. 2A; see also Table S3 in the supplemental material). Genes predicted to encode the lipopolysaccharide (LPS) core oligosaccharide are present immediately upstream of this locus (Fig. 2A). To determine if BACUNI_00969 is required for production of an O-ag, we adsorbed antiserum generated to whole-cell Bu8492 with the Bu8492 *tn::*00969 mutant. The antibodies remaining following this adsorption reacted with a small-molecular-size molecule of slightly more than 6 kDa (Fig. 2C), which is similar to the reported size of the LPS of Bu8492 and other *Bacteroides* species (31). To confirm that O-ag synthesis is abrogated in the Δ00969 mutant, we purified LPS by aqueous phenol extraction and examined it by silver staining and Western immunoblotting. Two major bands were observed for the LPS of wild-type (WT) Bu8492 indicative of a short, nonladdering O-ag (Fig. 2D). The band of highest molecular mass was strongly recognized by the adsorbed antiserum (Fig. 2D). The Δ00969 mutant lacks the higher-molecular-mass band and is no longer recognized by the antiserum. This higher-molecular-mass form was restored when BACUNI_00969 was added to the mutant in *trans* (Fig. 2D). Together, these data demonstrate that deletion of BACUNI_00969 abrogates O-ag biosynthesis and that production of this O-ag is required for BSAP-2 sensitivity.

### O-antigen loci in *B. uniformis* and proximity to the gene encoding BSAP-2.

**FIG 3:**
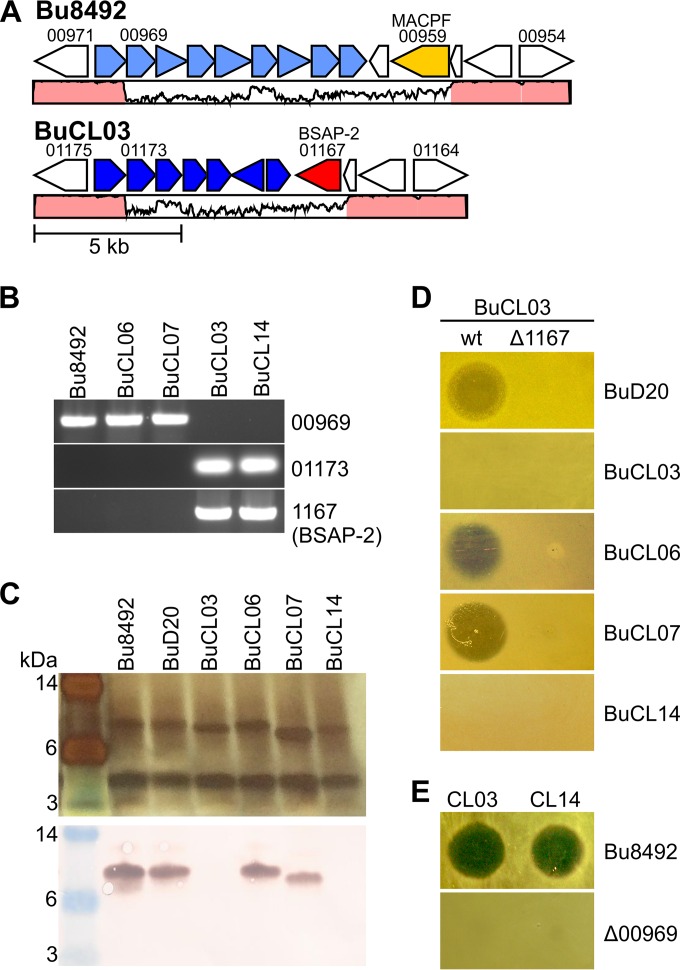
Correlation of O-ag with BSAP-2 in *B. uniformis* strains. (A) ORF maps showing the two types of O-ag loci (light and dark blue) in sequenced *B. uniformis* strains typified by Bu8492 and BuCL03. Surrounding genes are shown, including the BSAP-2-encoding gene colored red and a distinct MACPF domain protein-encoding gene colored yellow. Percent DNA identity is graphed below the open reading frames, with regions >95% identical colored in pink. (B) Ethidium bromide (EtBr)-stained agarose gel showing amplification products from various PCRs. Upper panel, Bu8492 O-ag region-specific gene; middle panel, BuCL03 O-ag region-specific gene; bottom panel, BSAP-2 gene. (C) Silver-stained gel and Western blot of purified LPS from various *B. uniformis* strains. The blot was probed with the adsorbed antiserum specific to the Bu8492 O-ag. (D) Agar overlays demonstrating the sensitivity or resistance of *B. uniformis* strains D20 (BuD20) CL03T00C23 (BuCL03), CL06T06C18 (BuCL06), CL07T00C16 (BuCL07), and CL14T09C07 (BuCL14) to BuCL03 or the BuCL03 mutant lacking the gene encoding BSAP-2. (E) Agar overlays showing zones of inhibition produced by BuCL03 and BuCL14 against Bu8492 but not the Bu8492 O-ag mutant (Δ00969).

BuCL03 is not targeted by BSAP-2, suggesting that this BSAP-2-producing strain must either lack an O-ag or have an O-ag distinct from that of sensitive strains. Comparison of the O-ag regions from *B. uniformis* genomes deposited in NCBI revealed that strains of this species have one of two distinct O-ag biosynthesis loci typified by Bu8492 (6 strains) or by BuCL03 (3 strains) (Fig. 3A; see also Fig. S3 in the supplemental material). The O-ag biosynthesis loci of Bu8492 and BuCL03 each begin with a conserved gene encoding a predicted glycosyltransferase (BACUNI_00970 and HMPREF1072_01174). The DNA then diverges, and each of the two loci encodes unique sets of predicted glycosyltransferases (see Table S3). Four *B. uniformis* strains from our collection were examined to determine if we could correlate O-ag type with BSAP-2 sensitivity or resistance. These strains included sequenced strain D20 (BuD20) and unsequenced human gut isolates CL06T06C18 (BuCL06), CL07T00C16 (BuCL07), and CL14T09C07 (BuCL14). BuD20 is one of the six sequenced *B. uniformis* strains that encode a Bu8492-like O-ag region. To determine if the unsequenced *B. uniformis* strains have an O-ag region similar to that of Bu8492 or BuCL03, we used PCR to amplify regions specific to each of these O-ag regions. A PCR product was amplified from the genomes of BuCL06 and BuCL07 using primers designed to correspond to the O-ag locus of Bu8492, and a product from BuCL14 was amplified using primers designed according to the O-ag locus of BuCL03 (Fig. 3B). We found that purified LPS from BuD20, BuCL06, and BuCL07 reacted with the antiserum to the O-ag of sensitive strain Bu8492 but that BuCL14 did not (Fig. 3C). In addition, the strains with a Bu8492-like O-ag were sensitive to BSAP-2 in agar overlays, whereas BuCL14, with a BuCL03-like O-ag, was not (Fig. 3D). These combined data show that *B. uniformis* strains have two major types of O-ag loci, one that correlates with BSAP-2 sensitivity and another that does not.

Our analysis of the O-ag region of these *B. uniformis* strains further revealed that the gene encoding BSAP-2 was located adjacent to the resistant O-ag locus in BuCL03 and other sequenced *B. uniformis* strains with a BuCL03-like O-ag region (Fig. 3A; see also Fig. S3 in the supplemental material). By PCR and agar overlay, we demonstrated that BuCL14 also contained the gene encoding BSAP-2 (Fig. 3B) and inhibited Bu8492 growth (Fig. 3E). These data strongly suggest that the gene encoding BSAP-2 was acquired along with the BSAP-2-resistant O-ag region. Interestingly, in four of the six *B. uniformis* strains that contain a Bu8492-like O-ag locus, there is also a gene encoding a protein with a MACPF domain (BACUNI_00959) directly adjacent to the O-ag locus. However, previous analysis of this protein did not reveal antimicrobial activity (25).

### An outer membrane protein of *B. fragilis* is the BSAP-1 target.

**FIG 4:**
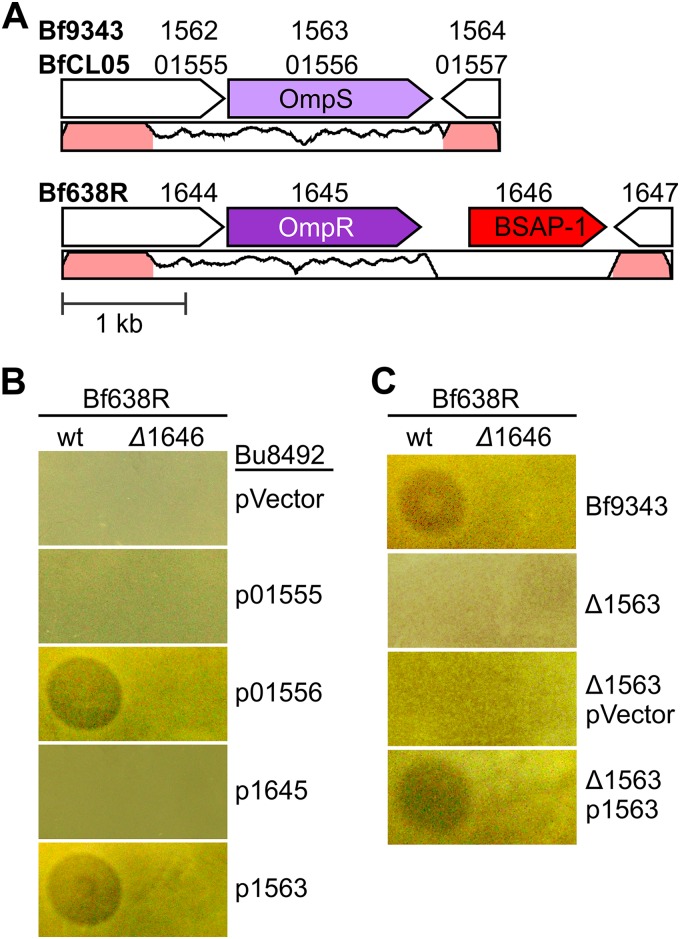
BSAP-1 targets an outer surface protein of sensitive *B. fragilis* strains. (A) Genetic region of heterogeneity between BSAP-1 producer Bf638R and BSAP-1-sensitive strains BfCL05T12C13 (BfCL05) and *B. fragilis* 9343 (Bf9343). The BSAP-1-encoding gene is colored red. The gene encoding the BSAP-1-targeted outer membrane protein (OmpS) is colored light purple, while its nontargeted ortholog in Bf638R (OmpR) is colored dark purple. Percent DNA identity between BfCL05 and Bf638R is graphed below open reading frames, and regions >95% identical are colored pink. (B and C) Agar overlays showing zones of inhibition produced by the Bf638R wild type (wt) or the BSAP-1 mutant (Δ1646) against Bu8492 with vector control (pVector), HMPREF1080_01555 (p01555), HMPREF1080_01556 (p01556), BF638R_1645 (p1645), or BF9343_1563 (p1563) (B) or against Bf9343, a clean deletion of BF9343_1563 (Δ1563), and Δ1563 with either the vector alone (pVector) or p1563 in *trans* (C).

On the basis of the finding that the gene encoding BSAP-2 is in the same genetic region as its target in sensitive strains, we attempted to identify the BSAP-1 target by analyzing the DNA region surrounding the gene encoding BSAP-1 (Fig. 4A)*.* We previously showed that among 56 *B. fragilis* strains analyzed, there were two predominant genetic types in this region: 21 strains harboring the gene encoding BSAP-1 and 35 strains without the gene encoding BSAP-1 (25). The region of heterogeneity between these two genetic types includes the gene encoding BSAP-1, a second upstream gene, and the 3′ region of a third gene (Fig. 4A). The upstream genes BF638R_1644 and HMPREF1080_01555 in strain Bf638R and sensitive strain BfCL05, respectively, encode proteins that are 85% similar to each other. The proteins encoded by immediate upstream genes BF638R_1645 and HMPREF1080_01556 of these same strains are 69% similar. All four of these proteins have signal I peptidase cleavage sites, indicating that they are exported from the cytoplasm. The proteins encoded by BF638R_1645 and HMPREF1080_01556 are predicted to be β-barrel outer membrane proteins of the porin superfamily.

To determine if either HMPREF1080_01555 or HMPREF1080_01556 of BfCL05 is the surface target of BSAP-1, we cloned the two individually and expressed them in the heterologous Bu8492 species (Fig. 4B). We found that expression of HMPREF1080_01556 but not expression of HMPREF1080_01555 was sufficient to confer BSAP-1 sensitivity to Bu8492. In contrast, expression of BF638R_1645, encoding the ortholog of HMPREF1080_01556, did not confer BSAP-1 sensitivity to Bu8492. To further confirm that this gene is required for killing of sensitive strains, we made a deletion mutant using BSAP-1-sensitive strain Bf9343 (ΔBF9343_1563), as strain BfCL05 is resistant to the antibiotics used for genetic manipulation of these strains. Deletion of BF9343_1563 abrogates the ability of BSAP-1 to target Bf9343, and BSAP-1 sensitivity is restored when BF9343_1563 is placed in *trans* (Fig. 4C). Thus, *B. fragilis* strains encode one of two similar porin-like outer membrane proteins, one that is targeted by BSAP-1 (OmpS) and one that is not (OmpR).

### BSAPs target surface molecules important for mammalian gut colonization.

**FIG 5:**
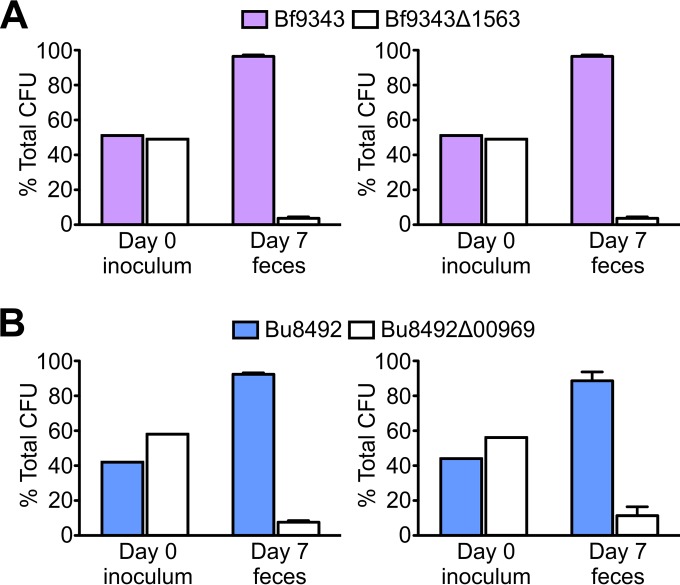
BSAP targets surface molecules critical for gut colonization. Data represent results of gnotobiotic mouse competitive gut colonization assays. (A) Bf9343 versus Bf9343Δ1563. (B) Bu8492 versus Bu8492Δ00969 (Bu8492ΔO-ag). Means and standard errors of the results of 2 separate trials of 3 mice each are graphed for each strain. Across both trials, the wt/mutant ratios in the inoculum compared to the day 7 feces samples were significantly different: for Bf9343 versus Bf9343Δ1563, *P* = 2 × 10^−6^; for Bu8492 versus Bu8492Δ00969, *P* = 3 × 10^−5^.

Our findings suggest that the BSAP surface targets and their orthologous or functionally equivalent molecules in BSAP-producing strains are important for these bacteria. First, if these surface molecules are expendable, their loss from BSAP-sensitive strains would be selected in natural competition with a BSAP-producing strain. In addition, the fact that BSAP-producing strains contain replacements rather than a deletion of these genes suggests an important function. To determine if mutants of BSAP surface targets are less fit than their WT counterparts, we first examined their growth rate in rich medium. Growth curves of Bf9343Δ1563 (Δ*ompR*) and Bu8492Δ00969 (ΔO-ag) are very similar to those of their wild-type counterparts (see Fig. S4 in the supplemental material). To test if these molecules are required for fitness *in vivo*, we performed competitive-colonization assays of the wild type and mutants using gnotobiotic mice (Fig. 5). We found that Bf9343Δ1563 lacking the BSAP-1-targeted OmpS had a significant colonization defect in comparison to Bf9343 (*P* = 2 × 10^−6^). Similarly, Bu8492Δ00969 lacking the BSAP-2-sensitive O-ag was outcompeted by wild-type Bu8492 (*P* = 3 × 10^−5^). We also examined the role of the BSAP-1-resistant ortholog OmpR in a background consisting of the Bf638R mutant lacking the gene encoding BSAP-1 (Δ1646) . Compared to the parental mutant, the Bf638R*Δ*1645Δ1646 mutant had a slight growth defect *in vitro* and displayed a significant colonization defect *in vivo* (*P* = 0.003) (see Fig. S4C and D). These data indicate that BSAP-1 and BSAP-2 surface targets are important for *in vivo* fitness, providing a biological rationale for the presence of these molecules in sensitive strains and the presence of orthologous surface molecules in BSAP-producing strains.

### BSAP-1 mediates competition in the mammalian gut.

**FIG 6:**
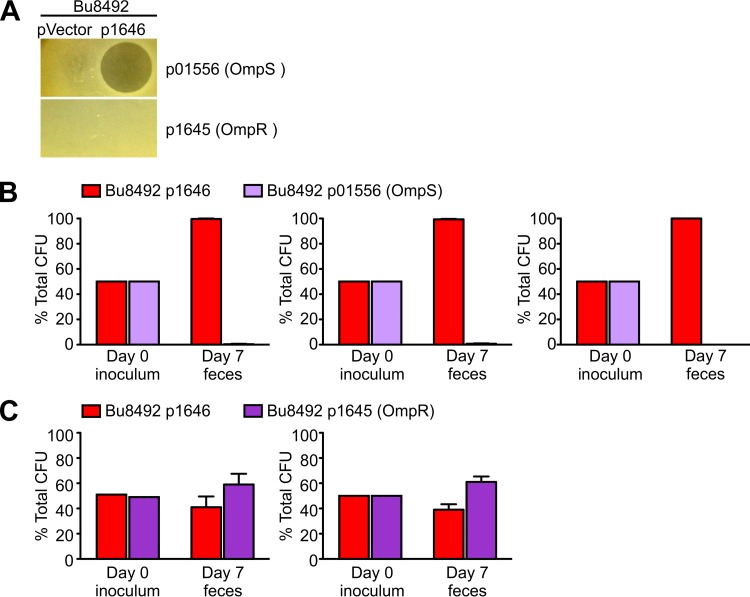
BSAP producers outcompete BSAP-sensitive strains in the mammalian gut. Isogenic strains that differed only in BSAP-1 production, sensitivity, or resistance were constructed by adding in to Bu8492 empty vector (pVector) or plasmids expressing BF638R_1646 (pBSAP-1), HMPREF1080_01556 (p01556), or BF638R_1645 (p1645). (A) Agar overlay assays of isogenic Bu8492 strains demonstrating BSAP production, sensitivity, and resistance *in vitro.* (B and C) Gnotobiotic mouse competitive-colonization assays between isogenic BSAP-1 producer and sensitive Omp (2 experiments, 3 mice each) (B) and BSAP-1 producer and resistant Omp (3 experiments, 3 mice each) (C). Means and standard errors are graphed. The ratios of strains in the inoculum versus day 7 feces samples were significantly different for BSAP-1 versus sensitive Omp (*P* = 1 × 10^−10^) but not for BSAP-1 versus resistant Omp (*P* > 0.05).

Next, we sought to determine if BSAPs provide a competitive advantage to the producing strain in the mammalian gut. We addressed this issue using BSAP-1, as only a single gene is necessary to confer BSAP-1 sensitivity whereas nine genes of the Bu8492 O-ag cluster are required to confer BSAP-2 sensitivity. We created an isogenic set of BSAP-1-producing, -sensitive, and -resistant strains so that we could attribute any fitness differences directly to BSAP-1. Genes encoding BSAP-1, the BSAP-1 target HMPREF1080_1556 (OmpS), and the BSAP-1-resistant ortholog BF638R_1645 (OmpR) were transferred to Bu8492. These isogenic Bu8492 strains, assayed in agar overlays, exhibited the expected phenotypes of BSAP-1 production, sensitivity, and resistance, respectively (Fig. 6A). Competitive cocolonization assays were performed by gavage of germfree mice with the BSAP-1 producer and an equal amount of either the BSAP-1-sensitive or BSAP-1-resistant isogenic strain. After 1 week, the BSAP-1-sensitive strain was outcompeted approximately 100-fold by the BSAP-1-producing strain (*P* = 1 × 10 ^− 10^) (Fig. 6B). In contrast, the ratio of the BSAP-1-resistant (OmpR) strain to the BSAP-1 producer strain in feces on day 7 was not significantly different from the ratios in the inoculums (*P* > 0.05) (Fig. 6C). These results demonstrate that BSAP production provides a fitness advantage to producers in competition with sensitive strains but not in competition with resistant strains.

### Cooccurrence analyses of BSAP-producing and -sensitive strains inferred from human gut metagenomics datasets.

The gnotobiotic mouse is a valuable experimental model to analyze competitive interactions between bacterial members in the mammalian gut. However, the ultimate goal is to understand the importance of these competitive interactions in the complex human gut ecosystem. An advantage of the gnotobiotic mouse model is that we are able to use isogenic strains and therefore can attribute any competitive effect directly to the BSAP. Human gut metagenomic datasets allow us to analyze natural populations and communities of bacteria, where numerous different cooperative and competitive interactions are at play. The combination of these analyses provides complementary data regarding the effects of BSAPs in the mammalian gut. We analyzed the 1,267 human gut metagenomes consolidated into the “3 consortium gene catalog” (3GCG) (32) to detect BSAP-producing strains cooccurring in the same human gut ecosystem with a BSAP-sensitive strain. Using translated datasets, we used a query cutoff of 95% protein identity over at least 20% of the protein to account for many short contigs in the metagenome assemblages. We detected BSAP-1 in 105 of 1,267 metagenomes, the BSAP-1 target HMPREF1080_01556 in 150, and the BSAP-1-resistant ortholog BF638R_1645 in 112 (see Table S4 in the supplemental material). BF638R_1645 was identified in 103 (98%) of the BSAP-1-positive metagenomes, which is consistent with their cooccurrence in sequenced *B. fragilis* genomes as expected. In contrast, BSAP-1 and its target, HMPREF1080_01556, do not cooccur in any human gut metagenomes, despite the fact that distinct *B. fragilis* strains can be present in the same human gut ecosystem (28, 33). This absence of cooccurrence differs significantly from the expected rate of cooccurrence under conditions in which BSAP-1 and HMPREF1080_01556 are independent (*P* = 2 × 10^−19^). These analyses suggest that *B. fragilis* strains producing BSAP-1 likely exclude sensitive strains in the human gut.

A similar analysis was performed to determine if BSAP-2 producers exclude sensitive *B. uniformis* strains. We searched the translated metagenomes for the presence of BSAP-2 and unique glycosyltransferases of the sensitive (BACUNI_00962 to BACUNI_00969) and resistant (HMPREF1072_01169 to HMPREF1072_01173) O-ag biosynthesis loci (see Table S5 in the supplemental material). To produce high-confidence inferences of O-ag type, we defined O-ag presence on the basis of identification of all unique glycosyltransferases encoded within the respective sensitive and resistant O-ag biosynthesis loci. BSAP-2 was identified in 179 metagenomes, and the five glycosyltransferases encoded by genes adjacent to the gene encoding BSAP-2 were found in 156 (87%) of those metagenomes. All of the genes encoding the eight glycosyltransferases unique to the BSAP-2-sensitive O-ag biosynthesis locus were found in 731 metagenomes, 57 (8%) of which also contained BSAP-2. This cooccurrence rate is only half of what would be expected if BSAP-2 and the sensitive O-ag genes were independently distributed in human gut metagenomes (*P* = 4 × 10^−14^). Therefore, *B. uniformis* strains producing BSAP-2 may exclude sensitive strains to some extent but with lower fidelity than strains producing BSAP-1. Together, these data indicate that BSAPs are ubiquitous in the human gut and that BSAP-1 production by *B. fragilis* may lead to strain exclusion, whereas BSAP-2-producing and -sensitive *B. uniformis* strains are able to cocolonize to some extent.

## DISCUSSION

In this report, we describe a mechanism of intraspecies interference competition among abundant *Bacteroides* species of the human intestinal microbiota. In both *B. fragilis* and *B. uniformis*, the BSAPs are MACPF domain proteins, which we have found to be ubiquitous in *Bacteroidetes* species, including species that occupy marine and soil habitats (25) (see Fig. S2 and Table S2 in the supplemental material). After discovering BSAP-1, we tested several *Bacteroides* MACPF domain proteins for antimicrobial activity and did not detect additional proteins with this activity (25). Therefore, it was unexpected that our random mutagenesis analysis performed to detect the BSAP of BuCL03 revealed another MACPF domain protein. As BSAP-1 and BSAP-2 are distinct and have little similarity, there are no sequence-based clues to help identify other likely candidates with antimicrobial activity among the diverse MACPF proteins of *Bacteroidetes*. The 322 MACPF domain proteins of *Bacteroidetes* identified here typically cluster by species, although there are a few exceptions, including those in cluster 4, where the genes encoding the 14 proteins of this cluster are found in 10 different *Bacteroidales* species (see Table S2). We previously showed that the gene encoding this protein is transferred between diverse *Bacteroidales* species on an integrated conjugative element (34). However, the restriction of most other MACPF proteins to a single species, or to two closely related species such as *B. vulgatus* and *B. dorei*, suggests that transfer of genes encoding MACPF domain proteins via integrative conjugative elements is not common.

The results of our gnotobiotic mouse studies indicate that the targets of BSAP-1 and BSAP-2 are necessary for *in vivo* fitness and thus that BSAP-resistant strains do not lack these molecules but instead encode a BSAP-resistant replacement molecule, likely with the same function. Despite their being in entirely different classes of molecules (proteins versus glycans) and being produced by different numbers of genes (one versus many), we found that both BSAP-1 and BSAP-2 are located adjacent to genes producing a resistant ortholog or functional equivalent of their cognate target molecules. This pattern suggests a general mechanism of BSAP acquisition in which BSAPs and genes encoding a resistant target are coacquired and in which acquisition is likely concomitant with loss of the gene encoding the BSAP-targeted surface molecule.

Two distinct mechanisms of interference competition in human gut *Bacteroidales* species have been described: contact-dependent type VI secretion systems (T6SSs) (19, 20) and secreted BSAPs (25). Genetic architecture 3 (GA3) T6SSs of *B. fragilis* (35), the only T6SSs of gut *Bacteroidales* shown to target bacteria, have a wide target range in that they kill numerous different gut *Bacteroidales* species (20) but are restricted to antagonizing those in which they make contact. In contrast, BSAPs have a narrow target range but are able to function at greater distance in that they are actively secreted from the cell. The combination of these characteristics suggests that the ecological role of BSAPs may be distinct from that of the T6SSs. Gnotobiotic mouse data (19, 20) and numerous human gut compositional studies have demonstrated that *B. fragilis* strains with GA3 T6SSs do not exclude sensitive *Bacteroidales* species in the gnotobiotic mouse intestine and the human gut. Therefore, the GA3 T6SSs likely function to create spatially structured niches for *B. fragilis*, restricting access of other *Bacteroidales* members. In contrast, BSAPs mediate intraspecies competition and thus function to antagonize strains that compete for similar niches. *B. fragilis* has been shown to preferentially colonize the mucus layer (36, 37) and to exhibit a preference for mucin glycans compared to dietary polysaccharides (38). Therefore, it is likely that distinct *B. fragilis* strains are in close proximity in the gut, where BSAPs would have a large impact on strain elimination, as we detected in human metagenomics datasets. *B. uniformis* bacteria have a greater ability to harvest nutrients from the diet based on the presence of a greater number of secreted glycoside hydrolases and polysaccharide lyases (13) and therefore may occupy diverse microniches of the gut. This may potentially explain the occasional cooccurrence of likely BSAP-2-producing and -sensitive *B. uniformis* strains in human gut ecosystems as determined on the basis of metagenomic data. The combination of analyses in this study, from genetic to functional to ecological, is beginning to allow us to decipher the competitive factors that are active in the human intestinal microbiota and their relevance to shaping and stabilizing this important health-related microbial community.

## MATERIALS AND METHODS

All primers used in this study are shown in Table S1 in the supplemental material.

### Bacterial strains and growth conditions.

*Bacteroides* strains analyzed in this study included *B. uniformis* ATCC 8492, *B. uniformis* CL03T00C23, *B. uniformis* D20, *B. uniformis* CL06T06C18, *B. uniformis* CL07T00C16, *B. uniformis* CL14T09C07, *B. fragilis* 638R, *B. fragilis* CL05T12C13, *B. fragilis* NCTC 9343, *B. fragilis* CM11, and *B. fragilis* CM13. *Bacteroides* strains were grown in supplemented basal medium (39) or on supplemented brain heart infusion (BHIS) plates. Antibiotics (5 µg/ml erythromycin or 3 µg/ml tetracycline) were added where appropriate. *Escherichia coli* strains were grown in L broth or L agar plates with the following antibiotics added where appropriate: ampicillin (100 µg/ml), trimethoprim (100 µg/ml), or kanamycin (50 µg/ml).

### Agar overlay assays.

Analysis of the ability of one strain to inhibit the growth of another by the secretion of inhibitory molecules was assayed using the agar spot test (40). *Bacteroides* strains were resuspended from a plate into phosphate-buffered saline (PBS), and 3-µl aliquots were spotted on a BHIS plate and grown anaerobically at 37°C overnight. The bacteria were removed with a swab or with Whatman filter paper, and the residual bacteria remaining on the plate were killed by exposure to chloroform vapor for 15 min. For overlays, 100 to 200 µl of logarithmically growing bacteria were mixed with 4 ml BHIS top agar (0.75% agar) and overlaid onto the chloroform-treated plate. The zones of inhibition were analyzed after overnight anaerobic incubation at 37°C.

### Transposon mutagenesis and insertion site identification in *B. uniformis* CL03T00C23 and *B. uniformis* 8492.

Random mutagenesis of *B. uniformis* CL03T00C23 and *B. uniformis* 8492 was performed using the transposon containing plasmid pYT646b (41). The transposon insertion site of the BuCL03T00C23 *tn*::1165 mutant was identified by cloning the junction, taking advantage of the β-lactamase gene and *E. coli* origin of replication contained at the end of the transposon. The chromosomal DNA was digested with HindIII followed by dilute ligation, transformed into *E. coli* DH5α, and plated on ampicillin plates. The junctional DNA from the resulting plasmid was identified by DNA sequencing using a primer directed out of the transposon. The transposon site of the Bu8492 *tn*::00969 mutant was identified by whole-genome sequencing.

### Creation of deletion mutants.

Internal nonpolar deletion mutants were constructed by amplifying DNA upstream and downstream of the gene or region to be deleted. PCR products were digested and cloned by three-way ligation into pNJR6 (42). The resulting plasmids were conjugally transferred into wild-type *Bacteroides* spp. using helper plasmid R751 (43), and cointegrates were selected by erythromycin resistance. Double cross-outs were screened by PCR for the mutant genotype.

### Cloning and heterologous expression of genes in *trans*.

Genes expressed in *trans* were PCR amplified and cloned into expression vector pMCL140 (44) or pFD340 (45). The resulting expression plasmids were verified by sequencing and transferred to *Bacteroides* strains by conjugal mating using helper plasmid RK231.

### Cloning and purification of His-1167.

HMPREF1072_01167 was PCR amplified so that the 21 N-terminal amino acids corresponding to the signal sequence were removed. The PCR product was digested with NdeI and BamHI and ligated into pET16b (Novagen), which produced a recombinant N-terminally His-tagged BSAP-2 fusion protein. Expression of the recombinant protein was induced with IPTG (isopropyl-β-d-thiogalactopyranoside), and the recombinant protein was purified using a ProBond purification system (Life Technologies) and the manufacturer’s recommendations for purification of native proteins. The eluted protein was dialyzed against PBS prior to analysis of activity.

### Western immunoblot analyses and silver staining.

Antiserum to whole-cell *B. uniformis* 8492 was prepared in rabbits by Lampire BioLogical Laboratories using the Express-Line polyclonal antiserum protocol. To create an antibody fraction specific to the molecule lost in *B. uniformis* 8492 *tn*::00969, we performed an antibody adsorption to remove antibodies reactive to the transposon mutant as previously described (46). For PAGE analysis, bacteria or purified LPS was boiled in lithium dodecyl sulfate (LDS) sample buffer and subjected to electrophoresis using NuPAGE 12% bis-Tris polyacrylamide gels with MES (morpholineethanesulfonic acid) buffer (Life Technologies). In some cases, the gel was subjected to silver staining (Pierce silver stain kit; Thermo Scientific) according to manufacturer protocols. For Western immunoblot analyses, the contents of the gels were transferred to polyvinylidene difluoride (PVDF) membranes and probed with the adsorbed serum. Alkaline phosphatase-labeled anti-rabbit IgG (Pierce) was the secondary antibody, and the membranes were developed with BCIP/NBT (5-bromo-4-chloro-3-indolylphosphate/Nitro Blue Tetrazolium) (KPL, Gaithersburg, MD).

### LPS extraction and silver staining.

LPS was purified by a modified aqueous phenol extraction method as previously described (47). Cultures (1.5 ml at an optical density at 600 nm [OD_600_] of 0.6) were centrifuged, and cells were resuspended in 200 µl of buffer containing 2% β-mercaptoethanol, 2% SDS, 10% glycerol, and 0.1 M Tris-HCl (pH 6.8). Samples were lysed by boiling for 15 min. Ten microliters of 20 mg/ml Pronase (Calbiochem) was added to lysates, and the reaction mixture was incubated overnight at 37°C. LPS was extracted from lysates by addition of 200 µl of Tris-saturated phenol, and the reaction mixture was incubated at 65°C for 15 min, followed by addition of 1 ml of ether. This mixture was subjected to vortex mixing and centrifuged, and the bottom aqueous fraction was collected. This extraction was repeated 3 times to yield purified LPS.

### Gnotobiotic mouse experiments.

Mouse studies were approved by the Harvard Medical Area Standing Committee on Animals. Swiss-Webster germ-free mice (4 to 6 weeks old) were obtained from the Harvard Digestive Diseases Center (HDDC) gnotobiotic facility and housed in sterile OptiMice cages (Animal Care Systems). Male mice and female mice were used in these experiments and were housed separately. Mice were colonized by gavage with the indicated strains mixed 1:1 (actual ratios shown in figures). After 7 days, fresh fecal samples from each mouse were collected, diluted in PBS, and plated for single colonies. Strains were differentiated and quantified by PCR by analyzing at least 90 colonies per mouse. For experiments using strains where genes were expressed from plasmids, 1 mg/ml of clindamycin was added to the drinking water to maintain the plasmids. A one-sample *t* test of arcsine transformed values was used to determine if the ratios of strains in the inoculum differed from those in feces.

### Identification of MACPF proteins encoded by *Bacteroidetes* genomes.

Protein sequences from bacteria within NCBI’s taxonomy database belonging to the division “CFB group bacteria” were searched for a MACPF domain as defined by Pfam. MACPF domain proteins were clustered at 99% amino acid identity over 100% of the sequence length, resulting in 149 clusters (see Table S2 in the supplemental material). A representative protein from each cluster was randomly selected and analyzed by the neighbor-joining method to create a bootstrap consensus tree based on 1,000 replicates (see Fig. S2 and Text S1 in the supplemental material).

### Analysis of *B. uniformis* O-ag biosynthesis loci.

Open reading frame (ORF) maps depicting *B. uniformis* genetic regions (see Fig. S3 in the supplemental material) were generated from GenBank files. Protein sequences of those ORFs were used as queries against the HMM database of Uniprot to generate multiple-sequence alignments (MSAs) using the HHblits program, with predicted secondary structures added to the MSAs by PsiPred. An HMM profile generated from each MSA was used as a query against profile-HMM databases, including CDD, COG, PDB, and Pfam. The results were compiled in Table S3 (see also Text S1).

### Analysis of human gut metagenomes.

Metagenomic analyses were conducted using 1,267 human gut metagenomes, representing a subset (3CGC) of a collection recently compiled without inclusion of the individually sequenced prokaryotic genomes (SPGC) (32). Amino acid sequences of complete and partial genes were compiled into a BLAST database, which was queried for proteins required for BSAP production, sensitivity, and resistance equivalents of BSAP targets. The output of these BLAST searches was parsed to include only hits reaching ≥95% identity over ≥20% query coverage and was compiled to produce Table S4 (BSAP-1) and Table S5 (BSAP-2) in the supplemental material. Cooccurrence relationships observed in human metagenomics data were assessed using the chi-square test for statistical independence. The expected rates of cooccurrence were defined as the product of the individual rates of occurrence. For more detail, see Text S1.

## SUPPLEMENTAL MATERIAL

Figure S1  Alignment of the MACPF domains of BSAP-1 and BSAP-2. Download Figure S1, PDF file, 0.04 MB

Figure S2  Phylogenetic tree of nonredundant *Bacteroidetes* MACPF proteins. Download Figure S2, PDF file, 0.4 MB

Figure S3  Genomic region of *B. uniformis* LPS core and O-antigen biosynthesis loci. Download Figure S3, PDF file, 0.04 MB

Figure S4  Role of BSAP target molecules and orthologs *in vitro* and *in vivo.* Download Figure S4, PDF file, 0.1 MB

Text S1  Supplemental experimental procedures and references. Download Text S1, PDF file, 0.1 MB

Table S1  Primers used in this study. Table S1, PDF file, 0.05 MB

Table S2  Clusters of *Bacteroidetes* MACPF domain-containing proteins. Table S2, XLSX file, 0.1 MB

Table S3  Predicted functions of *B. uniformis* proteins encoded by the O-antigen loci. Table S3, XLSX file, 0.2 MB

Table S4  Identification and cooccurrence of BSAP-1, OmpR, and OmpS (BSAP-1 target) in human gut metagenomes. Table S4, XLSX file, 0.1 MB

Table S5  Identification and cooccurrence of BSAP-2 and glycosyl transferases unique to the sensitive and resistant O-ag in human gut metagenomes. Table S5, XLSX file, 0.4 MB
