# Supplementary material for: Bacteroidales Secreted Antimicrobial Proteins Target Surface Molecules Necessary for Gut Colonization and Mediate Competition In Vivo
Source: mBio. 2016 Aug 23;7(4):e01055-16. doi: 10.1128/mBio.01055-16 (PMC4999547; doi:10.1128/mBio.01055-16)
Supplement: Figure S1 — Alignment of the MACPF domains of BSAP-1 and BSAP-2. Download [file mbo004162946sf1.pdf]

```
HMPREF1072_01167 - BSAP-2 ---LSANVSFDKESSNKTSITTKETYIRVDQIKQLERYQLYADPTVLADYLTPYFKSRLS 200
     Bf638R_1646 - BSAP-1 SGSLSSSFSSTQHYTSKYSIADYTSFI------RRRRLFLTASTELLSKYLTKMFVDDLS 174
                             **:..*  :. :.* **:   ::*      : .*  * *.  :*:.***  * . **

                   BSAP-2 TASPDAIIEEFGTHILIDFNVGGRLSIFYKSTITDNLKVESKTKIAKGGITGAIKTVNLS 260
                   BSAP-1 KQSPSFIIQHYGTHVLTDITLGGRITVLYRSSINTSKKTATV---EAGCASGIKNMFNLS 231
                          . **. **:.:***:* *:.:***:::*:*:*.  . *.  :      *  :*  : .***

                   BSAP-2 FSGSSTTTEVEQYQRRNSNWSCNVNMYGGQHDGH-TITITSDGATNHTFNLSGWQQSVDK 319
                   BSAP-1 VDGHYDQTLVKDNSEQE----IVYRTEGGDPSRALIGQLNYDSKNPSVIDISSWQQSCDD 287
                          ..*    * *:: ..::       .  **: .      :. *. . .:::*.**** *.

                   BSAP-2 THCVLTEINFNKTYPIYEFIKDPIKKQQIKDAAEKY 355
                   BSAP-1 NNMTLVDAEPGSLIPIYDLVSDMGKKEQLKLAVENY 323
                          .. .*.: :  .  ***:::.*  **:*:* *.*:*
```

Figure S1. Clustal-W alignment of the MACPF domains of BSAP-1 and BSAP-2

Amino acid sequence alignments of the MACPF domain of BSAP-2 (HMPREF1072_01167) and BSAP-1 (BF638R_1646) by Clustal-Omega (1). A conserved amino acid motif (Y/W-G-T/S-H-F/Y-X6-GG) of MACPF domains is boxed, including two glycine residues highlighted in yellow required for extension of the transmembrane beta hairpin (2). Residues of BSAP-1 previously shown by site-directed mutagenesis to be essential for functional activity (3) are highlighted in pink.

1. **Sievers F, Wilm A, Dineen D, Gibson TJ, Karplus K, Li W, Lopez R, McWilliam H, Remmert M, Soding J, Thompson JD, Higgins DG.** 2011. Fast, scalable generation of high-quality protein multiple sequence alignments using Clustal Omega. Mol Syst Biol **7:**539.
2. **Dunstone MA, Tweten RK.** 2012. Packing a punch: the mechanism of pore formation by cholesterol dependent cytolysins and membrane attack complex/perforin-like proteins. Curr Opin Struct Biol **22:**342-349.
3. **Chatzidaki-Livanis M, Coyne MJ, Comstock LE.** 2014. An antimicrobial protein of the gut symbiont Bacteroides fragilis with a MACPF domain of host immune proteins. Mol Microbiol **94:**1361-1374.
