# Supplementary material for: Bacteroidales Secreted Antimicrobial Proteins Target Surface Molecules Necessary for Gut Colonization and Mediate Competition In Vivo
Source: mBio. 2016 Aug 23;7(4):e01055-16. doi: 10.1128/mBio.01055-16 (PMC4999547; doi:10.1128/mBio.01055-16)
Supplement: Figure S2 — Phylogenetic tree of nonredundant Bacteroidetes MACPF proteins. Download [file mbo004162946sf2.pdf]

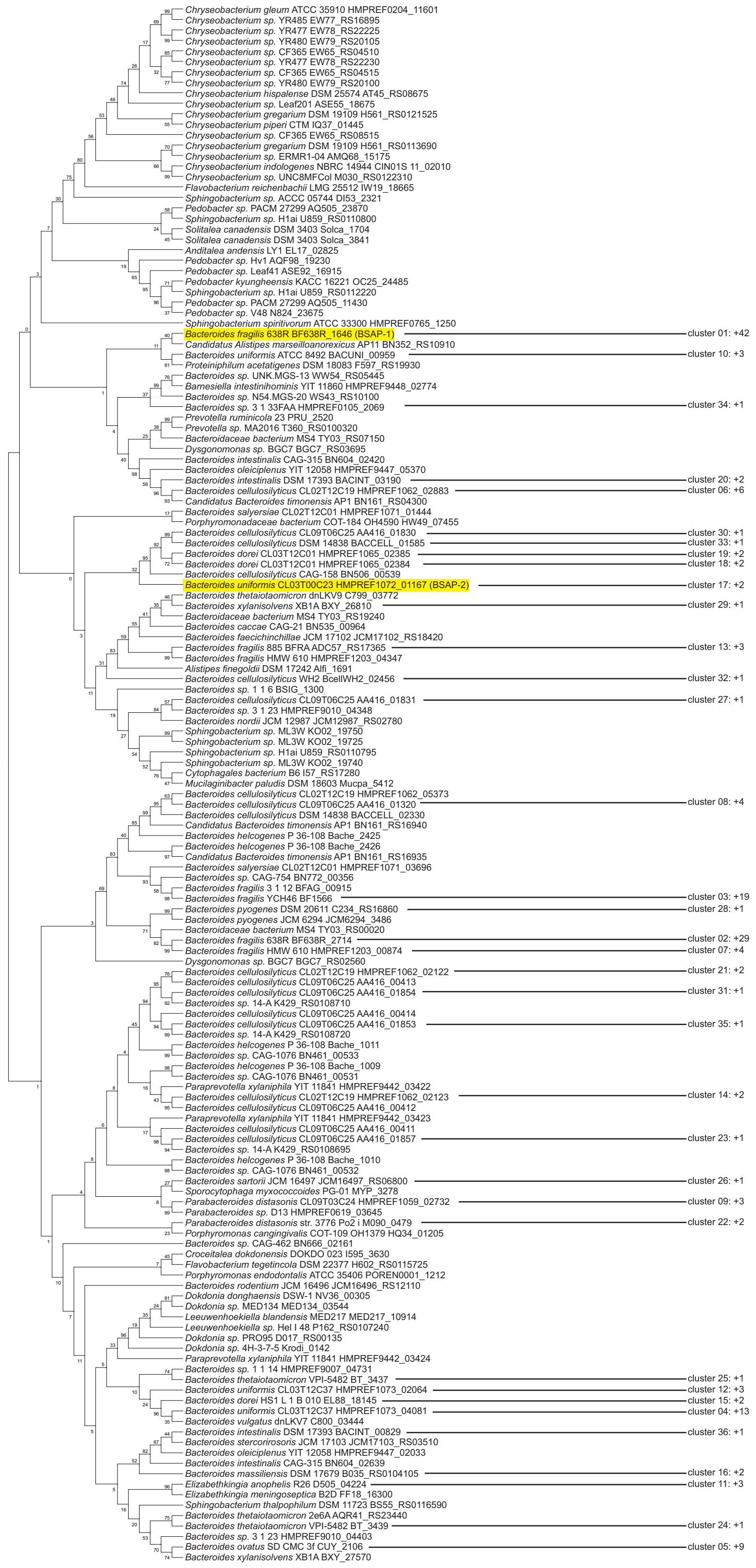

Figure S2. Phylogenetic Tree of non-redundant Bacteroidetes MACPF proteins

Phylogenetic tree of the MACPF domain proteins in Bacteroidetes species. BSAP-1 and BSAP-2 are highlighted in yellow. Only one protein is listed for each orthologous protein clustering at 99% similarity. Cluster number correlates with the list of strains/proteins in that cluster listed in Table S2. The number following the "+" designates how many other proteins fall into that cluster.
