# Supplementary material for: Bacteroidales Secreted Antimicrobial Proteins Target Surface Molecules Necessary for Gut Colonization and Mediate Competition In Vivo
Source: mBio. 2016 Aug 23;7(4):e01055-16. doi: 10.1128/mBio.01055-16 (PMC4999547; doi:10.1128/mBio.01055-16)
Supplement: Figure S3 — Genomic region of B. uniformis LPS core and O-antigen biosynthesis loci. Download [file mbo004162946sf3.pdf]

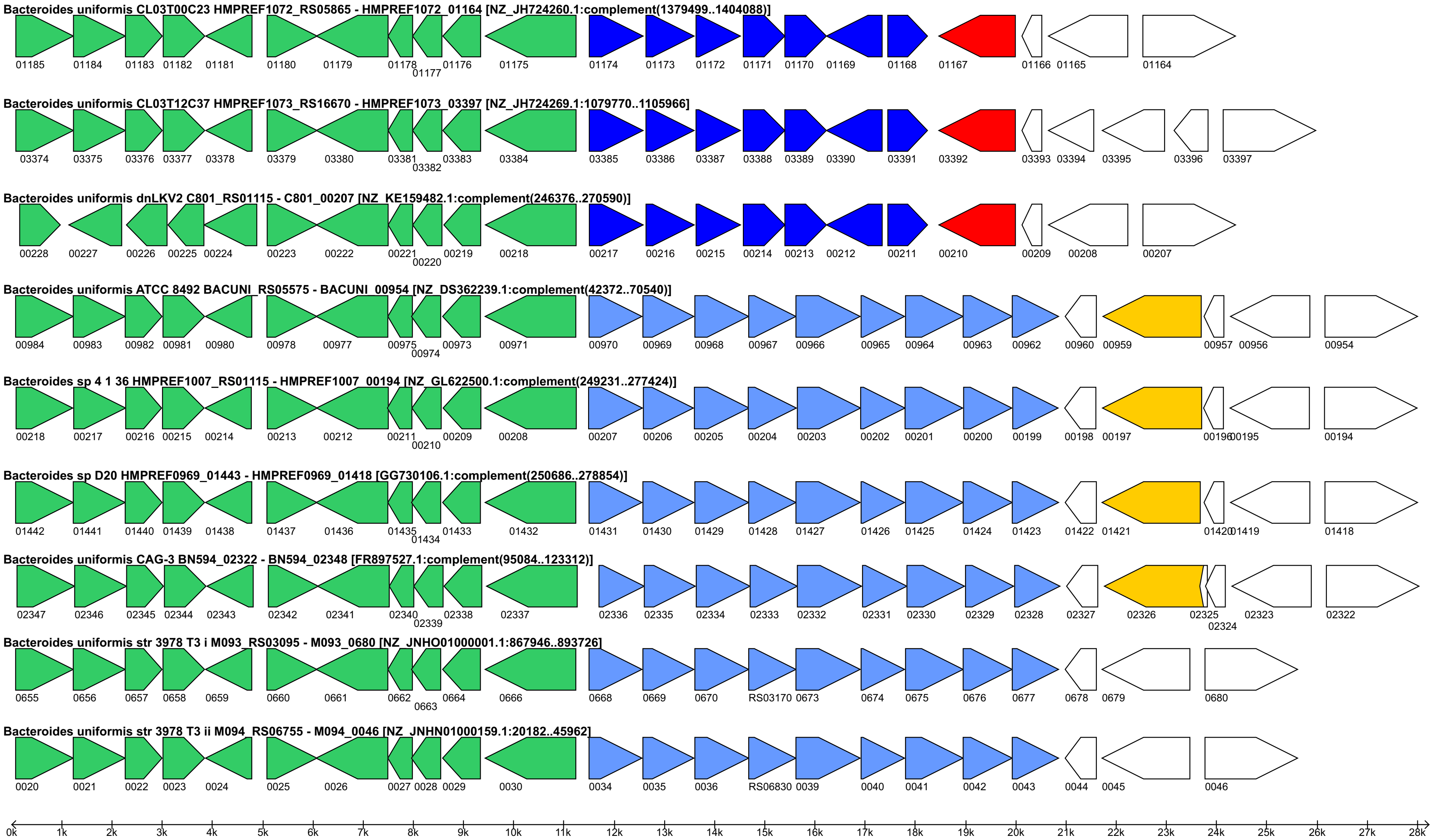

Figure S3. Genomic region of *B. uniformis* LPS and O-antigen biosynthesis loci

Predicted O-antigen biosynthesis genes are shown in blue for all sequenced *B. uniformis* strains showing that this region segregates into two major O-antigen locus types (light blue BSAP-2 sensitive, dark blue BSAP-2 resistant). Predicted LPS core glycan biosynthesis genes are colored green. Genes encoding BSAP-2 are colored red, and a second distinct MACPF domain protein, without antimicrobial activity, is indicated in yellow. For more information on predicted protein function, see Table S3.
