# Supplementary material for: Bacteroidales Secreted Antimicrobial Proteins Target Surface Molecules Necessary for Gut Colonization and Mediate Competition In Vivo
Source: mBio. 2016 Aug 23;7(4):e01055-16. doi: 10.1128/mBio.01055-16 (PMC4999547; doi:10.1128/mBio.01055-16)
Supplement: Figure S4 — Role of BSAP target molecules and orthologs in vitro and in vivo. Download [file mbo004162946sf4.pdf]

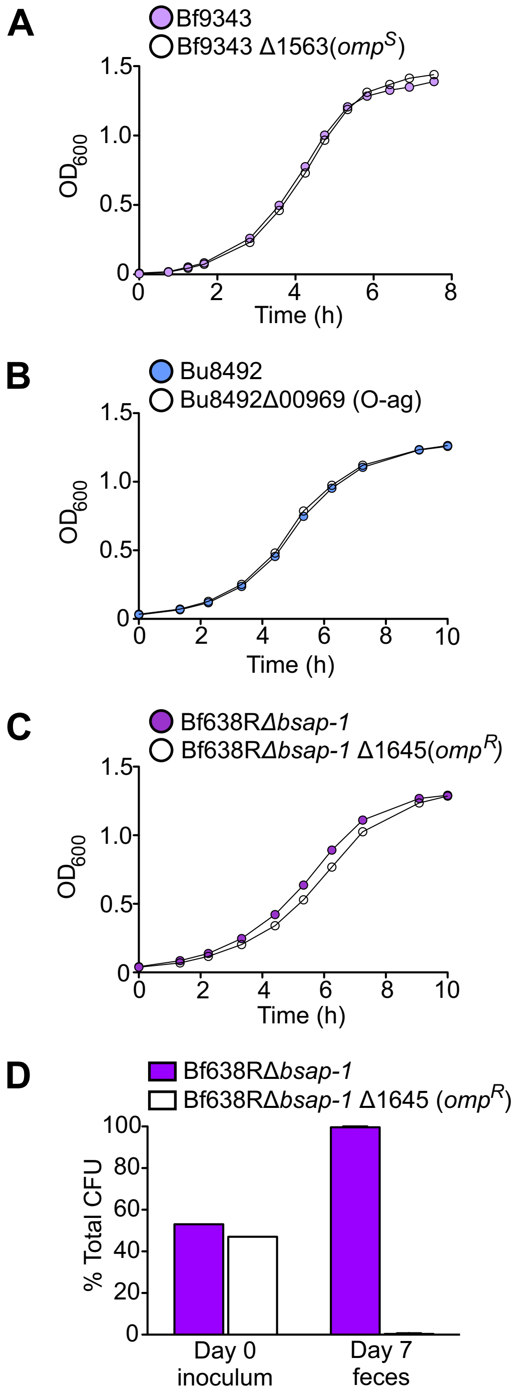

Figure S4. Role of BSAP target molecules and orthologs *in vitro* and *in vivo*

Growth curves of wild type and BSAP receptor or BSAP-resistant ortholog mutants measured by OD_{600} vs. time. Average and standard error of triplicate experiments are graphed. A) Bf9343 vs. Bf9343Δ1563 (Bf9343Δ*omp*^{S}), B) Bu8492 vs. Bu8492Δ00969 (Bu8492Δ*O-ag*), C) Bf638RΔ1646 (Bf638RΔ*bsap-1*) vs. Bf638RΔ1645-1646 (Bf638RΔ*bsap-1*Δ*omp*^{R}). D) Gut colonization competition assay of Bf638RΔ*bsap-1* vs. Bf638RΔ*bsap-1*Δ*omp*^{R}. 3 mice were used in this experiment. The ratio of strains in the inoculum and day 7 feces are significantly different, $p = 0.003$.
