# Supplementary material for: Bacteroidales Secreted Antimicrobial Proteins Target Surface Molecules Necessary for Gut Colonization and Mediate Competition In Vivo
Source: mBio. 2016 Aug 23;7(4):e01055-16. doi: 10.1128/mBio.01055-16 (PMC4999547; doi:10.1128/mBio.01055-16)
Supplement: Text S1 — Supplemental experimental procedures and references. Download [file mbo004162946s1.pdf]

Supplemental Experimental Procedures:

Identification of MACPF proteins encoded by Bacteroidetes genomes

All genomes identified in NCBI's taxonomy database as belonging to the division "CFB group bacteria" and that included a protein translation (.faa) file were downloaded from NCBI's FTP site on January 29, 2016. The resulting collection comprises 1,235 genomes and represents at least 4 classes, 19 families, 163 genera, and 376 species of the phylum Bacteroidetes. Using hmmsearch program (Linux version 3.1b2, February 2015; http://hmmer.org/), all 4,107,353 proteins encoded by these genomes were scanned for the presence of a MACPF domain using the profile hidden Markov model defined by Pfam (4) accession PF01823.15. As a result of this search, 322 proteins were identified from 208 genomes as having a full sequence bit score that equaled or exceeded the gathering threshold cut-off (21.50) for the profile HMM. This protein set was made non-redundant by clustering them at the 99% amino acid identity level over 100% of the length using the blastclust program provided in the stand-alone BLAST suite version 2.2.26 (5), resulting in 149 clusters, 36 of which contained two of more members, with the largest cluster containing 43 proteins (Table S2). A representative member of each cluster was pseudo-randomly selected (via Perl) and these 149 protein sequences were analyzed by the neighbor-joining method (6) using MEGA7 (7) to compile a bootstrap consensus tree (8) based on 1000 replicates shown in Fig S2.

Analysis of *B. uniformis* O-ag biosynthesis loci

Gene neighborhood analysis of nine *B. uniformis* genetic regions depicted in Fig. S3 were performed by downloading the sequences as GenBank (9) files and generating ORF maps of the regions. The protein sequences were also recovered from each GenBank file and each sequence was used as a query against the profile hidden Markov model (HMM) database Uniprot20 (dated June 20, 2015, downloaded from ftp://toolkit.genzentrum.lmu.de/pub/HH-suite/databases/hhsuite_dbs) to generate a multiple sequence alignment (MSA) with the HHblits program (64-bit version 2.0.16, compiled and run under CentOS 7 Linux, (10)), using three iterations and the default e-value cutoff of 0.001. Predicted secondary structure information added to each MSA by PsiPred (version 2.6, (11)). A profile HMM was generated from each MSA and used as a query against various profile-HMM databases, notably the CDD (Jan 14, 2015, (12)), COG (Jan 14, 2015, (13)), RCSB Protein Data Bank (PDB; Sep 12, 2015, (14)), and Pfam (version 28, (4)) profile databases (all profile databases were downloaded from ftp://ftp.tuebingen.mpg.de/pub/protevo/HHsearch/databases). Generally, the top three hits returned by each of these profile-profile searches for each of the *B. uniformis* proteins are shown in Table S3.

Analysis of human gut metagenomes

Metagenomic analyses were conducted using 1,267 human gut metagenomes, a subset (3CGC) of a collection recently compiled without including the individually sequenced prokaryotic genomes (SPGC) (15). The 159,325,886 amino acid

sequences of complete and partial genes from this set were downloaded from http://meta.genomics.cn and compiled into a BLAST database using the makeblastdb program from version 2.3.0 of the BLAST+ suite (16). For BSAP-1 analyses, this database was searched using as queries protein sequences BF638R_1645 (Omp^R) and BF638R_1646 (BSAP-1) of BSAP-1 producer *B. fragilis* 638R (GenBank accession FQ312004.1), and HMPREF1072_01556 (Omp^S) of BSAP-1 sensitive strain *B. fragilis* CL05T12C13 (Genbank accession JH724193.1). For BSAP-2 analyses, the database was searched using as queries 8 protein sequences (BACUNI_00962 - BACUNI_00969) corresponding to unique glycosyl transferases of the BSAP-2 sensitive O-antigen biosynthesis locus from *B. uniformis* ATCC 8492 (GenBank accession DS362217.1), as well as 5 protein sequences (HMPREF1072_01169 - HMPREF1072_01173) corresponding to unique glycosyl transferases from the BSAP-2 resistant O-antigen locus and HMPREF1072_01167 (BSAP-2) from *B.uniformis* CL03T00C23 (GenBank accession JH724260.1). The output of these BLAST searches were parsed to include only hits reaching ≥95% identity over ≥20% query coverage, and compiled to produce Table S4 (BSAP-1) and Table S5 (BSAP-2). Co-occurrence relationships observed in human metagenomics data were assessed using the chi-square test for statistical independence.

Supplemental References

1. **Sievers F, Wilm A, Dineen D, Gibson TJ, Karplus K, Li W, Lopez R, McWilliam H, Remmert M, Soding J, Thompson JD, Higgins DG.** 2011. Fast, scalable generation of high-quality protein multiple sequence alignments using Clustal Omega. Mol Syst Biol **7:**539.
2. **Dunstone MA, Tweten RK.** 2012. Packing a punch: the mechanism of pore formation by cholesterol dependent cytolysins and membrane attack complex/perforin-like proteins. Curr Opin Struct Biol **22:**342-349.
3. **Chatzidaki-Livanis M, Coyne MJ, Comstock LE.** 2014. An antimicrobial protein of the gut symbiont Bacteroides fragilis with a MACPF domain of host immune proteins. Mol Microbiol **94:**1361-1374.
4. **Punta M, Coggill PC, Eberhardt RY, Mistry J, Tate J, Boursnell C, Pang N, Forslund K, Ceric G, Clements J, Heger A, Holm L, Sonnhammer EL, Eddy SR, Bateman A, Finn RD.** 2012. The Pfam protein families database. Nucleic Acids Res **40:**D290-301.
5. **Ye J, McGinnis S, Madden TL.** 2006. BLAST: improvements for better sequence analysis. Nucleic Acids Res **34:**W6-9.
6. **Saitou N, Nei M.** 1987. The neighbor-joining method: a new method for reconstructing phylogenetic trees. Mol Biol Evol **4:**406-425.
7. **Kumar S, Stecher G, Tamura K.** 2016. MEGA7: Molecular Evolutionary Genetics Analysis Version 7.0 for Bigger Datasets. Mol Biol Evol doi:10.1093/molbev/msw054.
8. **Felsenstein J.** 1985. Confidence limits on phylogenies: An approach using the bootstrap. Evolution **39:**783-791.
9. **Benson DA, Cavanaugh M, Clark K, Karsch-Mizrachi I, Lipman DJ, Ostell J, Sayers EW.** 2013. GenBank. Nucleic Acids Res **41:**D36-42.
10. **Remmert M, Biegert A, Hauser A, Soding J.** 2012. HHblits: lightning-fast iterative protein sequence searching by HMM-HMM alignment. Nat Methods **9:**173-175.
11. **Jones DT.** 1999. Protein secondary structure prediction based on position-specific scoring matrices. J Mol Biol **292:**195-202.
12. **Marchler-Bauer A, Zheng C, Chitsaz F, Derbyshire MK, Geer LY, Geer RC, Gonzales NR, Gwadz M, Hurwitz DI, Lanczycki CJ, Lu F, Lu S, Marchler GH, Song JS, Thanki N, Yamashita RA, Zhang D, Bryant SH.** 2013. CDD: conserved domains and protein three-dimensional structure. Nucleic Acids Res **41:**D348-352.
13. **Tatusov RL, Fedorova ND, Jackson JD, Jacobs AR, Kiryutin B, Koonin EV, Krylov DM, Mazumder R, Mekhedov SL, Nikolskaya AN, Rao BS, Smirnov S, Sverdlov AV, Vasudevan S, Wolf YI, Yin JJ, Natale DA.** 2003. The COG database: an updated version includes eukaryotes. BMC Bioinformatics **4:**41.

14. **Berman HM, Westbrook J, Feng Z, Gililand G, Bhat TN, Weissig H, Shindyalov IN, Bourne PE.** 2000. The Protein Data Bank. Nucleic Acids Res **28:**235-242.
15. **Li J, Jia H, Cai X, Zhong H, Feng Q, Sunagawa S, Arumugam M, Kultima JR, Prifti E, Nielsen T, Juncker AS, Manichanh C, Chen B, Zhang W, Levenez F, Wang J, Xu X, Xiao L, Liang S, Zhang D, Zhang Z, Chen W, Zhao H, Al-Aama JY, Edris S, Yang H, Wang J, Hansen T, Nielsen HB, Brunak S, Kristiansen K, Guarner F, Pedersen O, Dore J, Ehrlich SD, Meta HITC, Bork P, Wang J, Meta HITC.** 2014. An integrated catalog of reference genes in the human gut microbiome. Nat Biotechnol **32:**834-841.
16. **Camacho C, Coulouris G, Avagyan V, Ma N, Papadopoulos J, Bealer K, Madden TL.** 2009. BLAST+: architecture and applications. BMC Bioinformatics **10:**421.
