# Supplementary material for: Bacteroidales Secreted Antimicrobial Proteins Target Surface Molecules Necessary for Gut Colonization and Mediate Competition In Vivo
Source: mBio. 2016 Aug 23;7(4):e01055-16. doi: 10.1128/mBio.01055-16 (PMC4999547; doi:10.1128/mBio.01055-16)
Supplement: Table S1 — Primers used in this study. [file mbo004162946st1.pdf]

Table S1. Primers

Organism, Target gene	Primer type	Sequence[a]
**Expression *in trans***		
***B. uniformis* CL03T00C23, HMPREF1072_01167**	**Forward**	5’-AAATGGATCCGTCCTTTCAAAAGGCAAACTAAAT-3’
	**Reverse**	5’-AATGGGATCCTGTTTCTGTTATGTCTTGGAGAATG-3’
***B. uniformis* 8492, BACUNI_00969**	**Forward**	5’-TTCGGGATCCCGGTTGTGGTAATTGTCATAGCTT-3’
	**Reverse**	5’-TTACGGATCCGCTATAACCCTTGCTTTATTCATT-3’
***B. fragilis* CL05T12C13, HMPREF1080_01555**	**Forward**	5’-AGGCGGATCCATTAAGAAACCAATAAACCACGGA-3’
	**Reverse**	5’-ATCGGGTACCTACCAACAGCCCCAAAGTTGCTA-3’
***B. fragilis* CL05T12C13, HMPREF1080_01556**	**Forward**	5’-AATAGGATCCCTCTTCAAGGGGCAGAAGATGATAA-3’
	**Reverse**	5’-GAAAGGTACCCGGGGATTTCTATACACACAAAAG-3’
***B. fragilis* 9343, Bf9343_1563**	**Forward**	5’-GGTAGGATCCTCTTCAAGGGGTAGAAGATGATAATGG-3’
	**Reverse**	5’-GAAAGGTACCCGGGGATTTCTATACACACAAAAG-3’
***B. fragilis* 638R, Bf638R_1645**	**Forward**	5’-GTGTGGATCCCGGTGGTCGAAGATAAGAAAATTGTG-3’
	**Reverse**	5’-TTTAGGTACCACTGCATCGTCCTTCAGGGATTT-3’
**His-BSAP-2**		
***B. uniformis* CL03T00C23, HMPREF1072_01167**	**Forward**	5’- TTTGCATATGACCAACACTTTTGACGAGCAGTTTG-3’
	**Reverse**	5’-AATGGGATCCTGTTTCTGTTATGTCTTGGAGAATG-3’
**Deletion mutants**		
***B. uniformis* CL03T00C23, ΔHMPREF1072_01165-01167**	**Upstream flank 5’**	5’-CGAAGGATCCTAGTCATAACAGCCTAGCACAAGC-3’
	**Upstream flank 3’**	5’-TTAGACGCGTCAAGCAATGAACAGAAGTACGATT-3’
	**Downstream flank 5’**	5’-TAAAACGCGTCCGTAGAGTATGCCAAAGCTAAAT-3’
	**Downstream flank 3’**	5’-CGTTGGATCCTGTGAACAATCAGGATAAAATTGC-3’
***B. uniformis* CL03T00C23, ΔHMPREF1072_01167**	**Upstream flank 5’**	5’-TTCAGGATCCCCTAAATGCCCACATTCTCTACTT-3’
	**Upstream flank 3’**	5’-AAAACCATGGCCCATTGTAATGATGGGAATAAAA-3’
	**Downstream flank 5’**	5’-TAAACCATGGCCGTAGAGTATGCCAAAGCTAAAT-3’
	**Downstream flank 3’**	5’-GCTGGGATCCTATAATTCTTTCAGATGCTGGTCG-3’
***B. uniformis* 8492, ΔBACUNI_00969**	**Upstream flank 5’**	5’-GATAGGATCCCTGGTTGATGAGGTCATAGCATAG-3’
	**Upstream flank 3’**	5’-CTCTACGCGTTCAGAGGTAGTGTCTCAGTGTTCTC-3’
	**Downstream flank 5’**	5’-GGCAACGCGTAGGACATTCTTCAAATCTAAACGG-3’
	**Downstream flank 3’**	5’-GCAGGGATCCCTGAATAATATCAAATCCACGACG-3’
***B. fragilis* 9343, ΔBf9343_1563**	**Upstream flank 5’**	5’-CTGAGGATCCTCTTTGCCTAAACCTGAAGAAGTC-3’
	**Upstream flank 3’**	5’-TAATGCGGCCGCAGCCCCAAAGTTGCTAAAGTAAT-3’
	**Downstream flank 5’**	5’-TGCCGCGGCCGCACAACCAAAGTGACTAATACCCGT-3’
	**Downstream flank 3’**	5’-TGAGGGATCCCCGGATGAAAGATGTAGAAGAGT-3’
***B. fragilis* 638R,**	**Upstream flank 5’**	5’-CGATGGATCCTTGTCCAGAAATTTTTAAAGACGAT-3’

**ΔBf638R_1645-1646**	**Upstream flank 3'**	5'-ATGCACGCGTTGCAGTCCATAAAAGAACTAATGC-3'
	**Downstream flank 5'**	5'-AGTAACGCGTTTGGGGCTAATGGAAATAGAAGTA-3'
	**Downstream flank 3'**	5'-GGGAGGATCCATTGAACAAGTATTTTGAAGGGGA-3'

[a]Restriction sites in primer sequences are underlined.
